# Qualification and Registration

**Published:** 1906-09-01

**Authors:** 


### Qualification and Registration.

The first thing a medical student has to do is to register himself as such, because the five years of study which are necessary in order to obtain a qualification are dated from the time of registration. For this purpose application must be made to the Registrar of the General Medical Council, 299 Oxford Street, London, W.

The student, however, cannot be registered until he has (1) passed a preliminary examination in Arts which is recognised by the General Medical Council, and (2) has commenced medical study.

A complete list of the recognised preliminary examinations may be obtained on application to the Registrar of the General Medical Council; and it is essential in any case to discover at a sufficiently early period what preliminary examinations are recognised by the particular examining body that it is proposed to work under. For example, with few exceptions, the London Matriculation is the only preliminary examination which will permit a candidate to proceed to the higher examinations in order to become a graduate of the University of London.

Wherever he proposes to study and whatever qualification he intends to get, the courses of study which a medical student has to undergo are really very much the same; they may be divided, generally speaking, into the two groups of the preliminary and the clinical.

But it is not, as a rule, compulsory to undergo the necessary courses of instruction in these two divisions of medical education at the same school. Often a student will be well advised to undergo his preliminary training at a provincial school, or as a student of the University of London, and to pursue his clinical studies elsewhere, as in one of the large Metropolitan hospitals.

The following is a list of the examining bodies and the degrees and diplomas granted by them which admit the graduate or diplomate to enter his name in the register of legally qualified medical men:—

ENGLAND.

| Examining Body. | Qualification. |
|---|---|
| University of Oxford ... | M.B., B.Ch. (This degree is only obtainable after graduating in arts.) |
| University of Cambridge ... | M.B., B.C. |
| University of London ... | M.B. |
| University of Durham ... | M.B. |
| University of Birmingham ... | M.B., B.Ch. |
| University of Liverpool ... | M.B., Ch.B. |
| Victoria University of Manchester ... ... ... | M.B., Ch.B. |
| University of Leeds ... ... | M.B., Ch.B. |
| University of Sheffield ... | M.B., Ch.B. |
| Conjoint Examining Board in England ... ... ... | M.R.C.S., L.R.C.P. |
| Society of Apothecaries of London ... ... ... | L.S.A. |

SCOTLAND.

| Examining Body. | Qualification. |
|---|---|
| University of Edinburgh ... | M.B., Ch.B. |
| University of Glasgow ... | M.B., Ch.B. |
| University of Aberdeen ... | M.B., Ch.B. |
| University of St. Andrews ... | M.B., Ch.B. |
| Conjoint Examining Board in Scotland | L.R.C.S.Edin., L.R.C.P. Edin., & L.F.P.S.Glasg. |

IRELAND.

| | |
|---|---|
| University of Dublin ... ... | M.B., B.Ch., B.A.O. (These degrees are only conferred on graduates in arts.) |
| Royal University of Ireland... | M.B., B.Ch. |
| Conjoint Examining Board in Ireland ... ... ... | L.R.C.P.I., L.R.C.S.I. |
| Apothecaries' Hall of Ireland | L.A.H.Dub. |

Many of these examining bodies lay down certain restrictions as to residence, and it is therefore important that an intending candidate should learn what these restrictions are by applying to the examining body from which he wishes to obtain his qualification.

### MEDICAL QUALIFICATIONS FOR WOMEN.

Women are admitted to degrees or diplomas by all English, Scotch, and Irish Universities (Oxford and Cambridge excepted). The Apothecaries' Society, London, the Conjoint Boards of England, Scotland, and Ireland.

Medical schools for women only are the School of Medicine for Women, 8 Hunter Street, Brunswick Square, London; the Medical College for Women, Edinburgh; and Queen Margaret College, Glasgow. Women are also admitted to schools of medicine in connection with the Universities of Dublin, Durham, Liverpool, Manchester, Birmingham, Leeds, Sheffield, and Aberdeen; the Catholic University, Dublin; Bristol University College, and also to special classes at the School of Medicine of the Royal Colleges, Edinburgh; the Schools of Surgery of the Royal College of Surgeons in Ireland; and of the Queen's Colleges of Belfast, Cork, and Galway. Students of the University of Glasgow receive their education at Queen Margaret College, which is an integral part of the University. Two years only of the medical curriculum can be taken at the United College, St. Andrews; the remaining three years are taken at University College, Dundee, where the whole five years can be passed if desired. Women can also attend classes for the first three years of the medical curriculum at University College, Cardiff.

